# Acceptability of Peer Support for People With Schizophrenia in Chennai, India: A Cross Sectional Study Amongst People With Lived Experience, Caregivers, and Mental Health Professionals

**DOI:** 10.3389/fpsyt.2022.797427

**Published:** 2022-03-08

**Authors:** Sonia Sims, S. Hepsipa Omega Juliet, Jainey Joseph, Subhashini Gopal, Vijaya Raghavan, Lakshmi Venkatraman, Ramachandran Padmavati

**Affiliations:** Department of Psychosocial Rehabilitation, Schizophrenia Research Foundation, Chennai, India

**Keywords:** schizophrenia, peer support, peer support volunteer, psychosocial intervention, peer support program, acceptability

## Abstract

**Introduction:**

Establishing structured peer support in mental health, particularly for people with schizophrenia, as a psychosocial intervention is early in low and middle-income countries like India. Before implementing and understanding the effectiveness of peer support service and which mode of peer support delivery will be suitable for our culture, our study aimed to understand if peer support would be accepted by the different participants like persons with schizophrenia, caregivers and mental health professionals in a tertiary care center in Chennai, India.

**Methods:**

The study was conducted at the outpatient department (OPD) of a tertiary psychiatric care facility in Chennai, India. A cross-sectional study method was used. Consecutive persons diagnosed with schizophrenia and caregivers of persons with schizophrenia, who attended the outpatient department, and mental health professionals within and outside the facility who met the inclusion and exclusion criteria participated in the study. A structured questionnaire purposefully developed for the study was administered to the different study participants. Descriptive statistics were used to analyze the data. Categorical variables were expressed as frequency and percentages, while the continuous variables were expressed as mean and standard deviation.

**Results:**

A total of 155 participants (52 persons with schizophrenia, 50 caregivers and 53 mental health professionals) completed the survey. The majority of the participants (90.4% of persons with schizophrenia, 86% caregivers and all mental health professionals) welcomed peer support interventions. The participants wanted peers to help persons with schizophrenia achieve personal goals to enhance their mental health and day to day living with an emphasis on independent living and interpersonal and social relationships and help them achieve medication and treatment-related goals toward recovery. Understanding the role of a peer support volunteer and transitioning from a “person with schizophrenia” to a “peer support volunteer” by persons with schizophrenia was thought most challenging.

**Conclusion:**

The results highlight the potential acceptability of peer support across several stakeholders in the care of schizophrenia in a low and middle-income country context. The results may guide the implementation of a peer support volunteer programme as an essential mechanism of delivering psychosocial interventions for persons with schizophrenia.

## Introduction

Peer support can be broadly defined as a process through which people who have equal standing with another and share everyday experiences or face similar challenges come together as equals to give and receive help based on the knowledge that comes through shared experience (1). A Peer Support Volunteer (PSV) in a mental health setting would refer to a person who has a lived experience of mental ill health.

Peer support in mental health has been gaining focus, indicating that peer support has the potential to drive through recovery-focused changes in services and can transform both individuals and systems (2). Research on peer support across several populations in developed countries have demonstrated benefits in better coping with experiencing a sense of connectedness, or group belonging, positive opportunities of sharing personal stories (3), cordial relationship between peers and non-peer staff, self-efficacy resulting from the experience of helping others (4), increased wellness secondary to working (5) and earning money (6).

Peer support signifies the importance of personal interests and strengths as foundations to recovery, rather than psychopathology and treatment considerations (7). Studies have also reported several challenges and unforeseen risks, including exposure to misleading information, facing hostile or derogatory comments from others, or feeling more uncertain about one's health condition (3). Peer support workers can also face adverse experiences, which also include non-peer staff discrimination and prejudice, difficulty managing the transition from “patient” to “peer support worker” and having a poor understanding of the role of a “peer support worker.” However, these potential risks can further be managed or controlled through training, supervision and attention given to solution-focused strategies (5).

The concept of peer support has received little attention in low- and middle-income countries (LMICs) such as India. Research on peer support workers' role in delivering evidence-based mental health interventions is still in the early stages (8). There are few efficacy and effectiveness studies on peer support and fewer policies to support personal recovery programmes (9). Peer support can be an asset for LMICs as it is less expensive and adds value to the mental health workforce (10).

There are very few studies on peer support in India, and have been from one part of the country only. As part of the Quality Rights project in Gujarat, India (11, 12), peer support volunteers (PSVs) were trained in recovery-oriented care and basic communication skills. In addition to working toward personal goals, they also created a professional employment space for peers (9). Other researchers who examined these trained PSVs in the mental health clinical setting reported that peer support had yielded positive outcomes (13). However, there is a need to explore this knowledge in other parts of the country to help develop peer-support programs localized to different Indian settings and cultures, as it will be complementary to the existing mental health services available to persons with mental illness disorders.

In keeping with this need, our research aimed to study the acceptability of peer support delivered by persons with schizophrenia (PWS) to other PWS by interviewing different stakeholders, including PWS, caregivers and Mental Health Professionals (MHP) in a tertiary care psychiatric facility.

The study objectives were to understand potential acceptability, the expectation of roles, feasibility of peer support and expected challenges from the different participant perspectives.

## Materials and Methods

### Study Site and Context

The study was conducted at the outpatient department (OPD) of a tertiary psychiatric care facility in Chennai, India. This facility caters to persons with mental disorders from Chennai in the southern state of Tamil Nadu in India. The facility also receives referrals from surrounding areas, other districts of Tamil Nadu and nearby states. Comprehensive mental health services that include pharmacological, psychological and psychosocial treatments to persons with various mental illnesses are offered as part of the clinical services.

Psychosocial treatments and interventions are provided through the department of psychosocial rehabilitation by a dedicated and experienced team of MHP, including psychiatrists, psychologists, and psychiatric social workers. During the group training and enhancement programs, a naturally occurring informal peer support initiative between PWS has been observed, indicating the need to examine this phenomenon systematically.

### Study Design

A cross-sectional study design was used. Institutional ethics committee (IEC) approval was obtained before the start of the study (SRF-CR/08/MAR-2020).

### Study Participants

All persons with an established diagnosis of schizophrenia and clinically stable in the age group 18 years and above, caregivers (family members primarily responsible for caring for PWS) and MHP (with at least 3 years' experience in providing care to PWS) who were willing and able to provide written informed consent were included in the study. The exclusion criteria were PWS and caregivers on their first visit to the facility and those who refused to consent for the study.

### Data Collection

Consecutive persons diagnosed with schizophrenia and caregivers of PWS who attended the outpatient department over 3 months, and MHP within and outside the facility, who met the inclusion and exclusion criteria, were included in the study. As the study period was interrupted by COVID second wave and lockdown restrictions, the data collection was extended for a further 4 month period to allow enough number of patients and caregivers to be included in the study. After obtaining written informed consent, the questionnaire was administered by the trained researchers. Data were collected between February and August 2021.

### Assessments

A structured proforma was used to collect the essential sociodemographic variables such as age, gender, residential area, and others from the stakeholders.

For this study, the following operational definition of peer-support and peer-support volunteer was used while explaining peer-support among the participants. Peer support was defined as a system of giving and receiving help founded on fundamental principles of respect, shared responsibility and mutual agreement of what is helpful (14). Peer support volunteer was defined as “a person who has a living experience of schizophrenia and is willing to support persons with similar life experiences and challenges faced”.

A structured survey questionnaire (version 1) was first developed for the study to explore the objectives of the study based on the review of literature and discussion amongst the research team. The questionnaire was then piloted amongst a small group of participants, and the items were modified based on feedback. The finalized questionnaire (version 2) consisted of 11 items (9 items were asked of all participants, one additional question was asked to PWS, and two additional questions were asked to MHP). Supplementary Table 1. This was used to collect data from the participants. The questions included acceptance of peer support, roles and services expected from a PSV, anticipated challenges and logistics of peer support service delivery. In addition, the MHP were asked for their opinion about supervision for the PSVs, and the PWS were asked if they would be interested in taking up the role of PSV. The questions had multiple options for the participant to choose from. “Other” option was provided for open ended responses that were not already covered in the multiple options.

### Data Analysis

The data analysis was done using SPSS 20.0.after cleaning and quality check and excluding missing data. Descriptive statistics were used to analyze the data. Categorical variables were expressed as frequency and percentages, while the continuous variables were expressed as mean and standard deviation.

## Results

One hundred and fifty-five participants (52 PWS, 50 caregivers, and 53 MHP) were interviewed for the study. Eight PWS refused to participate in the study. The open-ended responses to the questions were a very small number and hence they were not taken up for analysis.

### Sociodemographic Profile of the Participants

Table 1 describes the sociodemographic characteristics of the participants.

**Table 1:** The socio-demographic characteristics of the stakeholders.

	**Persons with schizophrenia** **(*n* = 52)**	**Caregivers** **(*n* = 50)**	**Mental health professionals** **(*n* = 53)**
Age Mean (sd)	43 (11.4)	50.1 (13.6)	34.3 (7.1)
Duration of illness			
Mean (sd)	13.4 (8.3)	NA	NA
**Sex**			
Males *n* (%)	31 (59.62%)	22 (44%)	16 (30.2%)
Females *n* (%)	21 (40.38%)	28 (56%)	37 (69.8%)
**Place of stay**			
Urban *n* (%) Rural *n* (%)	45 (86.54%) 7 (13.46%)	45(90%) 5(10%)	NA
Experience in working with PWS in years Mean (sd)	NA	NA	8.9 (6.3)

The mean age of the PWS included in the study was 43 (sd 11.4) with an age range from 19 to 72 years. The mean duration of illness of PWS was 13.4 (sd 8.3). There were more male PWS 31(59.62%) who participated in the study. The majority [45(86.54%)] belonged to the urban background.

Caregivers were on an average −50.1(sd 13.6) years, and 32(64%) were either parents or spouses. There were more female 28(56%) than male 22(44%) caregivers, and 45(90%) of the caregivers were from an urban background.

The mean age of the MHP included in the study was 34.3 (sd 7.1). Most, 33(60%) of the MHP interviewed were psychiatric social workers. There were more female 37(69.8%) MHPs than males 16(30.2%).

### Acceptance of Peer Support

Forty-seven (90.4%) PWS, 43(86%) caregivers, and 53(100%) MHP supported the idea of peer support, as shown in Table 2. Forty-four (84.6%) PWS were willing to share information about their life events, illness, treatment, side effects, and personal information about themselves and their family with the PSV. Thirty-one (59.6%) PWS were willing to become PSV themselves. Forty-two (84%) caregivers were willing for PWS to share information to PSV regarding day-to-day happenings, challenges, and experiences faced. Fifty-two (98.1%) MHPs felt that PWS could share information regarding the illness and other aspects depending on their comfort level.

**Table 2:** The acceptance of various stakeholders of peer support volunteers.

	**Persons with schizophrenia (*n* = 52)**	**Caregiver of individuals with schizophrenia (*n* = 50)**	**Mental health professional (*n* = 53)**
Acceptance toward peer support in dealing with problems			
YES *n* (%) NO *n* (%)	**47(90.4)** 5(9.6)	**43(86)** 7(14)	**53(100)** 0(0)
Acceptance of sharing information with peers support volunteers			
YES *n* (%) NO *n* (%)	**44(84.6)** 8(15.4)	**42(84)** 8(16)	**52(98.1)** 1(1.9)
Willing to be a peer support volunteer			
YES *n* (%) NO *n* (%)	**31(59.6)** 21(40.4)	nil	

*The bold values represent highest values, highest preferences*.

Those who did not want peer support stated reasons of being unsure of how one PWS can help another PWS as a peer support volunteer, and with good family support, there was no need for peer support. Those who did not want to share information with PSV cited confidentiality and privacy as the reasons.

### Types of Support Expected From PSV

As shown in Table 3, more than two thirds, 37(71.2%) PWS wanted peers to help them achieve better mental health by providing them with emotional and psychological support when it is required, listen and understand problems, help them deal with day-to-day challenges and share positive experiences. 35(67.3%) PWS wanted peers to help them achieve independent living by providing them with motivation and encouragement to take responsibility toward a particular aspect of life, build a routine, and help them avail welfare benefits.

**Table 3:** The types of support expected from peer support volunteers.

	**Persons with schizophrenia** **(*n* = 52)**	**Caregiver of individuals with schizophrenia** **(*n* = 50)**	**Mental health professional** **(*n* = 53)**
Better mental health *n* (%)	**37(71.2)**	**34(68)**	35(66)
Independent living *n* (%)	**35(67.3)**	32(64)	37(69.8)
Interpersonal and social relationships *n* (%)	30(57.7)	**34(68)**	**40(75.5)**
Achieve overall goals *n* (%)	30(57.7)	24(48)	25(47.2)
Medication and treatment support *n* (%)	29(55.8)	28(56)	**46(86.8)**
Employment *n* (%)	29(55.8)	25(50)	35(66)

*The bold values represent highest values, highest preferences*.

Caregivers stated expectations from peers in helping PWS achieve better mental health 34(68%) and good interpersonal and social relationships 34(68%) by providing them companionship and helping PWS socialize.

The majority of the MHPs 46(86.8%) wanted PSV to be involved in helping PWS in medication and treatment-related support where peers can educate PWS on illness and treatment, ensure adherence, accompany PWS to the clinic if required, build trust and help them feel accepted by helping them know that they are not alone in the struggle toward living with mental illness. MHP also had an expectation of peers helping PWS achieve interpersonal and social relationships 40(75.5%) and independent living 37(69.8%). All stakeholders, 29(55.8%) PWS, 25(50%) caregivers, 35(66%) MHPs, expected less support from peers in employment-related aspects.

### Mode of Delivery of Peer Support

Around half the participants, 26(50%) PWS, 26(52%) caregivers, 27(50.9%) MHP reported that the peers could be of any gender. Thirty (57.7%) PWS, 28(56%) caregivers, 21(39.6%) MHP reported that peers could be of any age. Forty-four (88.6%) PWS, 40(80%) caregivers, 26(49.1%) MHP reported peers could be of any religion and 29(55.8%) PWS, 36(72%) caregivers, 46(86.8%) MHP stated that PSV should speak the same language as the PWS.

One to one peer support by meeting face to face was preferred by 42 (80.8%) PWS, 37 (74%) caregivers and 52 (98.1%) MHP and group peer support by meeting face to face was preferred by 31(59.6 %) PWS, 32(64%) caregivers and 46(86%) MHP. Participants were also open to other modes of interaction between PSV and PWS, such as telephonic contact, which was preferred by 26(50%) PWS, 26(52 %) caregivers and 37(69.8 %) MHP and text messaging was preferred by 18(34.6%) PWS, 17(34.7%) caregivers and 24(45.3%) MHP. Contact made through social media platforms was the least preferred mode of contact among all participants (6(11%) PWS, 4(8.2 %) caregivers, 5(9.4 %) MHP).

Most, 36(69.2%) PWS and 31(62%) caregivers stated that the frequency of contact should be as and when required, MHPs on the other hand, preferred planned contacts with the PSV on weekly 33(62.3%) or monthly 29(54.7%) basis.

The majority of the participants, 37(71.2 %) of PWS, 36(72%) caregivers, and 37(69%) MHP, preferred mental health professionals to choose the PSV for the PWS, followed by choices of PWS and peer volunteers. The involvement of caregivers in choosing the PSV for the PWS was comparatively less preferred amongst all the participants. Almost 51(96.2%) of the MHP stated that peers ought to be supervised by mental health professionals.

### Potential Challenges in Delivering Peer-Support

Majority of the MHP [(43) 81.1%] stated that the patient's understanding of the role of a PSV can be difficult, and 35 (66%) also stated that transitioning from the role of a “person with schizophrenia” to a “peer support volunteer” may be most challenging. Several other challenges noted were poor sustainability 31(58.5%), the possibility of a negative relationship being created 31(58.5%), discrimination 30(56.6%), facing unfriendly critical comments 28(52.8%), and stigma 28(52.8%). Eleven (20.8%) MHPs stated other challenges such as over-familiarity, lack of boundaries, risk of transference between the PSV and PWS, caregiver interference, and the possibility of the PSV feeling burdened with the workload and having difficulty in coping with the stress, which could lead to a relapse.

Caregivers were concerned about PSV ability to understand the mental state of others, misguidance and unhealthy relationships being challenges. PWS stated that interpersonal issues such as disagreements, misunderstandings between peers and PWS, privacy and confidentiality could be challenging in peer support.

## Discussion

This study attempted to explore the attitudes and opinions toward peer support volunteer work in a tertiary mental health setting. The findings indicate that a majority of the interviewed participants, which included PWS, their caregivers and MHPs, were willing to accept peer support, could perceive its benefits and were willing to utilize such services. Participants' willingness to embrace the peer support program is crucial to implementing and integrating it within the existing clinical service (15). About half the PWS were unwilling to become PSVs themselves. This may have been due to poor confidence and lack of clarity on what being a PSV entails. Training and providing well-defined structure to roles of PSV may help the PSV get an understanding of the scope of their work (16) and provide them the confidence to take on this role.

Whilst the study in Gujarat has noted how peers had taken on roles that identified themselves as “service providers” (13), PWS and caregivers this study have expressed a consensus for PSV in taking up more informal roles as “companion,” with whom PWS can feel connected, share information, be supported and motivated toward achieving personal goals such as better mental health, independent living and building up their interpersonal and social relationships.

This study has shown that majority of the MHP expect PSV to be involved in improving medication and treatment adherence of PWS. This is in keeping with the largely medical model of mental illness adopted by MHP both in training and practice (17).

All the participants do not expect support in the area of employment from PSV, the reasons for these needs to be explored further. The above expectations need to be kept in mind while developing a peer support programme that suits the local population. The needs of PWS should be matched to a peer's willingness to take on specific roles.

Paid peer work has emerged as an employment pathway for people in recovery (18). However, studies have also highlighted “issues of power” where there is a possibility of power imbalances in paid work. There is also the risk of peers over-professionalizing their work and the “dark side of peer support.” Employed as a peer worker may cause a risk for future employment opportunities, tension around the workforce, risks regarding how peer workers access mental health services for their health, risk of peer workers experiencing trauma or re-traumatization through peer work and risks of bringing peer work and peer workers into the mainstream service system (19). Finding a steady funding source to support paid PSV can be an issue. Peer support is still in its early stages of development in India. No previous literature is available in the area of paid peer volunteers and their impact, but the Quality Rights project in Gujarat paid the PSVs a small honorarium to cover their travel and expenses (~US$50 per month) supported by project funds initially followed by the provision of financial resources by the State Mental Health Authority, Government of Gujarat since June 2016 (9). It will be essential to educate the managers and policymakers and provide training at the service level to all the stakeholders to overcome these barriers. Future research needs to explore whether peers should be paid for the services they are willing to provide.

India being a diverse country, it was essential to understand the socio cultural factors that might be important when planning peer support. Interestingly all the participants emphasized language when matching PSV to PWS and placed lesser emphasis on socio cultural factors such as gender, age and religion.

While participants have stated that peers can connect with PWS using any mode of interaction, most participants preferred one-to-one, face-to-face interactions, followed by face-to-face group interactions. Participants felt that in-person interactions are more personal than teleconsultation, and it is much easier to observe emotional cues, talk about emotional issues and offer appropriate support, as noted in other literature (20). Technology is, however, increasingly being applied to deliver peer support to individuals with mental health conditions (21). Similarly, in the current study, participants were also open to telephonic contact. Social media was least preferred by all participants who stated that PWS might have less knowledge and usage of social media platforms, and it can be a risk to privacy and confidentiality. The majority of PWS and caregivers preferred frequency of contact to be as and when required as they felt PWS could communicate with peers based on need, crisis and urgency, but MHP preferred a more planned contact being established between peer and PWS so that there is a lesser chance of peers being overwhelmed or burdened. Future peer support programmes could focus on finding a middle ground between planned and anytime contact within a set of boundaries.

Even though peer support focuses on empowerment (22–24), the PWS and caregivers have stated preference toward the MHP to be the one to choose the PSV for the PWS. This reliance on MHP can be interpreted as lack of empowerment of PWS or a higher level of PWS and caregivers' trust in the MHP. It has been noted that MHP often select peers based upon their communication skills, understanding of mental illness, personal responsibility, compassion and level of clinical stability, and professionals are ones to supervise peers during the delivery of support services (25).

In keeping with earlier research (16) this study also shows that majority of the challenges perceived are concerning the PWS understanding of the role of the PSV and that PWS may have difficulty in transitioning from “PWS” to “peer,” although this transition is seen as a gradual process. While developing a programme for PSV, it is vital to ensure that PSV has role clarity and training and supervision to ensure PWS can positively transform into a PSV and maintain the integrity of peer support, such that peer support workers are seen as peers and not para-professionals (26).

This study, establishes that PSV programme is acceptable and necessary in our setting. Further studies are needed to explore the benefits of peer support not only for the recipients of mental health services, but also for the PSV and the mental health care system as a whole. The feasibility and maintenance of a robust PSV programme in health care would only be possible through collaborative efforts and ongoing support and engagement from all stakeholders (27). PSV are more than professionally qualified at promoting recovery outcomes such as hope, empowerment, self-esteem and self-efficacy, social inclusion, and engagement (2, 9, 28).

Several limitations undermine the generalizability of the findings.

The small sample size and lack of a planned and justified sample size of each stakeholder group restricts meaningful comparison between the groups. The study participants are not representative of the geography of the country. The severity of the illness of the PWS, which could impact the decision-making capacity in taking part in peer support, was not explored. The study was conducted in a tertiary psychiatric setting by mental health professionals who possibly reinforced medical assumptions around peer support. Since this study is the first time exploring peer support volunteers undertaken in this part of the country and it is a relatively new concept that is yet to be introduced, a cross-sectional study design using a structured survey questionnaire was used. Future research could fill this gap and adopt a mixed-method design with qualitative exploration.

In conclusion, this study throws light on PSV being a potential resource for mental health delivery. The data provides evidence that training peers can add to the country's limited workforce resources for delivering mental health care. The future steps will be to explore in depth what existing peer support model would suit the local needs and adapt and implement a model that can then be evaluated for its effectiveness.

## Data Availability Statement

The raw data supporting the conclusions of this article will be made available by the authors, without undue reservation.

## Ethics Statement

The studies involving human participants were reviewed and approved by Institutional Ethics Committee of SCARF. The patients/participants provided their written informed consent to participate in this study.

## Author Contributions

SS, SG, VR, LV, and RP contributed to the conception and design of the study. SS organized the database and wrote the first draft of the manuscript. VR performed the statistical analysis. SS, VR, SH, and JJ wrote sections of the manuscript. All authors contributed to manuscript revision, read, and approved the submitted version.

## Conflict of Interest

The authors declare that the research was conducted in the absence of any commercial or financial relationships that could be construed as a potential conflict of interest.

## Publisher's Note

All claims expressed in this article are solely those of the authors and do not necessarily represent those of their affiliated organizations, or those of the publisher, the editors and the reviewers. Any product that may be evaluated in this article, or claim that may be made by its manufacturer, is not guaranteed or endorsed by the publisher.

## Abbreviations

PWS: Persons with Schizophrenia
PSV: Peer Support Volunteer
MHP: Mental Health Professionals
LMIC: Low- and Middle-Income Countries.
