# Supplementary material for: Acceptability of Peer Support for People With Schizophrenia in Chennai, India: A Cross Sectional Study Amongst People With Lived Experience, Caregivers, and Mental Health Professionals
Source: Front Psychiatry. 2022 Mar 8;13:797427. doi: 10.3389/fpsyt.2022.797427 (PMC8965369; doi:10.3389/fpsyt.2022.797427)
Supplement: Supplementary file 1 [file Data_Sheet_1.docx]

**Supplementary Material**

**Semi structured questionnaire used among the study participants**

A peer can simply be defined as someone who has an equal standing with another, and most of the time is of the similar age group sharing similar interests and status in life. (Merriam-Webster Dictionary)

**Peer support definition**:  Mead (2003) defines peer support as, ‘a system of giving and receiving help founded on key principles of respect, shared responsibility and mutual agreement of what is helpful’. Synergistic understanding that benefits both parties

**Description of a peer volunteer in mental health:** a peer volunteer is a person who has a living experience of Schizophrenia and is willing to support people with similar life experiences and challenges faced.

**Demographic details of Person with Schizophrenia (PWS)**

1. Participant ID:
2. Age:
3. Gender:
4. Diagnosis:
5. Duration of illness:
6. Place of Stay: Urban / Rural

**Demographic details of caregiver**

1. Participant ID:
2. Age of the caregiver:
3. Gender of the caregiver:
4. Relationship of the caregiver to the patient:
5. Place of stay: urban/ rural
6. Are you primarily and largely responsible for providing care to your relative with Schizophrenia? Yes/ No

**Demographic details of mental health professional(MHP)**

1.    Participant ID:

2.    Age :

3.    Gender:

4. Mental health professional occupation: Psychiatrist/ Psychiatric Social worker/ Psychologist/ Occupational therapist/ Others _____________

5.    Work experience with PWS in years_________________

**A.**   **I would like to ask you some questions about your opinion on “peer support volunteers”(PSV) to help persons with schizophrenia (PWS).**

1. Would you like PWS to have peer support in dealing with patient’s problems? YES or NO
2. Would you be comfortable towards PWS sharing information with a peer (another PWS)? YES or NO

If yes, what information­­____________________________________________

1. Do you think a peer support worker for the PWS should be

a)    Gender:  same/ different / any/no comment

b)    Age: same/ different / any /no comment

c)    Language: same/ different/ any/ no comment

d)    Religion: same/ different/ any/no comment

**B.**   **Now I would like to ask you some specific questions about peer support work and peer support volunteers in helping PWS. You can identify more than one option in each item, as applicable.**

1. Do you think peer support should be delivered to the PWS in

a)    Face to face in group form

b)    Face to face in one-to-one basis

c)    Through the telephone

d)    Through social media (such as Facebook etc)

e)    Don’t know

f)     Others describe ___________________

g)    No comment

5.    What would you like the PSV to do for the PWS?

a)    Give friendly company

b)    Provide emotional support

c)    Motivate them

d)    Listen to them and share positive experiences

e)    Listen and understand patient’s problems

f)     Help them to deal with day to day challenges

g)    Be available and approachable when the patient is in need

h)    Make PWS responsible in a particular aspect of life

i)     Help PWS in work related aspects

j)     Help PWS to make friends

k)    Helping PWS to avail govt benefits or welfare benefits

l)     Others please mention __________________________

m)  No comment

6.    What would a PSV help PWS achieve?

a)    Patient’s overall goals

b)    Help achieve independent living

c)    Help with interpersonal and social relationships

d)    Help with employment

e)    Help patient with medication and treatment related support

f)     Help to improve better mental health

g)    Others ________________

h)    No comment

7.    How do you think the PWS can associate with a peer?

a)    Someone chosen by mental health professional

b)    Someone patient chooses

c)    Someone chosen by the caregiver

d)    Someone who volunteers to help patient

e)    More than one of the above

f)     Any other________________________

g)    No comment

8.    At what frequency do you think PWS should meet with a peer

a)    Several times a week

b)    Weekly

c)    Fortnightly

d)    Monthly

e)    As and when required

f)     Others _________________________

g)    No comment

9.    What problems do you think can occur between PSV and PSW? (this question was asked as an open ended question to PWS and Caregivers, the below options were given to the MHP)

a)    Facing unfriendly or critical comments

b)    Stigma

c)    Discrimination

d)    Difficulty managing the transition from “PWS” to “PSV”

e)    Having a poor understanding of the  role of a “PSV”

f)     Negative relationship

g)    Poor sustainability in the peer support programme

h)    Others please mention_______________________________

i)     None

j)     No comment

Other comments_____________________________________________

**PWS were additionally asked**

10. Would you like to become a peer support volunteer? YES/NO

**Mental Health Professionals were additionally asked**

  10. Do you think peer support volunteers need supervision YES/ NO?

  11. If stated yes to supervision, who do you think should supervise a PSV?

a)    Caregivers

b)   Mental health professionals

c)    None

d)   Others ________________

  12. Do you think peer support is required in our set up? YES/ NO
